# Supplementary material for: A Novel Antibody-Drug Conjugate Targeting Nectin-2 Suppresses Ovarian Cancer Progression in Mouse Xenograft Models
Source: Int J Mol Sci. 2022 Oct 15;23(20):12358. doi: 10.3390/ijms232012358 (PMC9604294; doi:10.3390/ijms232012358)
Supplement: Supplementary file 1 [file ijms-23-12358-s001.zip › ijms-1912605-supplementary.pdf]

Supporting Information

Table S1. Primer sequences used for qRT-PCR

Genes	Primer sequences
GAPDH	Forward: 5′-GGGTGTGAACCATGAGAAGTATGAC-3′<br>Reverse: 5′-GTCCTTCCACGATACCAAAGTTGTC-3′
NECTIN-2	Forward: 5′-CCAGAAGGTCACGTTCAGCC-3′<br>Reverse: 5′-CAGTCCAGGGATGAGAGCCA-3′

Table S2. si-RNA Sequences used for nectin-2 knock-down experiment

**Names**	**si-RNA sequences**
#1	Sense: 5′-UGACCUGGCUCAGAGUCAUAGCCAA-3′<br>Antisense: 5′-UUGGCUAUGACUCUGAGCCAGGUCA-3′
#2	Sense: 5′- CCUGAUACCUGUGACCCUCUCUGUA-3′<br>Antisense: 5′- UACAGAGAGGGUCACAGGUAUCAGG-3′
#3	Sense: 5′- CACCUUCGUCUGCACAGUCACCAAU-3′<br>Antisense: 5′- AUUGGUGACUGUGCAGACGAAGGUG-3′

Table S3. IC_{50} values of tested materials

Cell lines	c12G1 (nM)	c12G1-DM1 (nM)	c12G1-MMAE (nM)
OV-90	N.D*	0.1	0.3
SK-OV-3	N.D	7.4	19.6
Caov-3	N.D	3.5	5.9
Daudi	N.D	33.6	20

*N.D: not determined up to 200 μg/ml

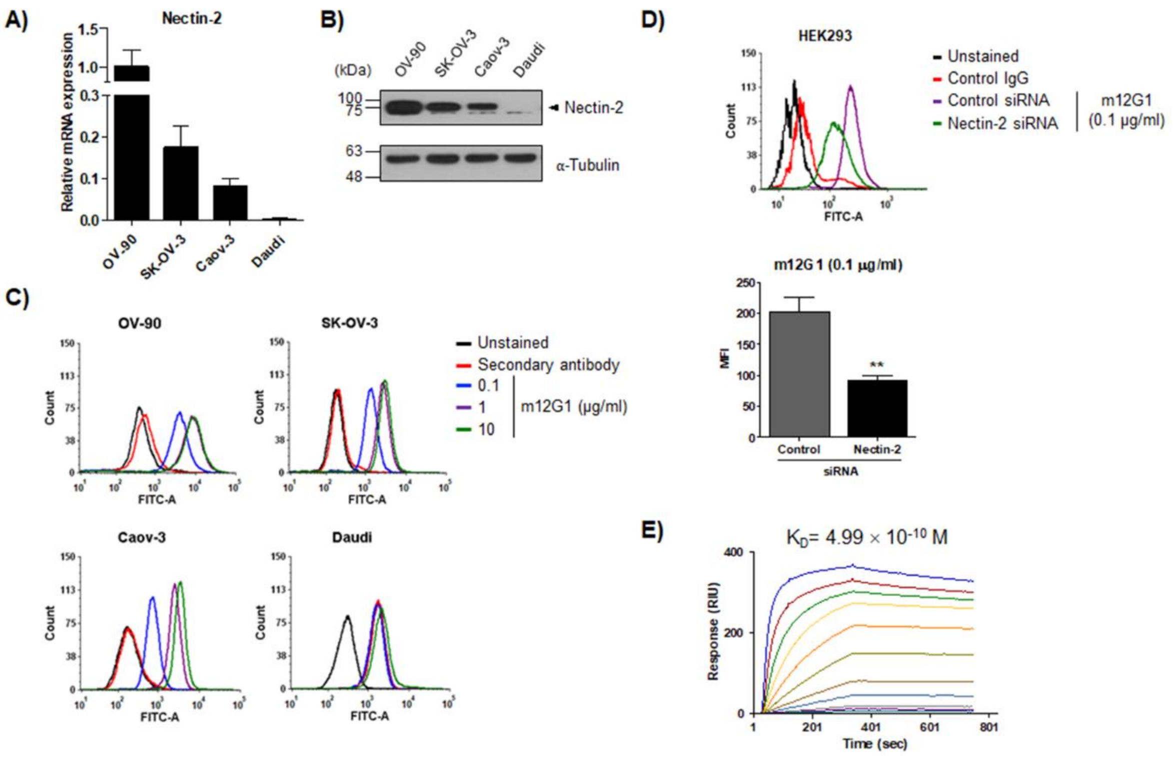

Figure S1. Characterization of 12G1 antibody (m12G1). Nectin-2 expression was examined using qRT-PCR (A) and western blot (B). Daudi cells were used as nectin-2 negative cell line. (C) Ovarian cancer cells were treated with the indicated concentration of m12G1 antibody and the binding of m12G1 antibody was analyzed by FACS. (D) The binding specificity of m12G1 antibody was examined by FACS analysis using si-RNA knock-down. HEK293 cells were transfected with 40 nM of control or nectin-2 si-RNA for 72 h. Then, FACS analysis was carried out as described in the Methods section (**, vs. control si-RNA). (E) The binding affinity of m12G1 antibody to human nectin-2 was examined using SPR analysis. All experiments were independently repeated at least three times. ** $P < 0.01$.

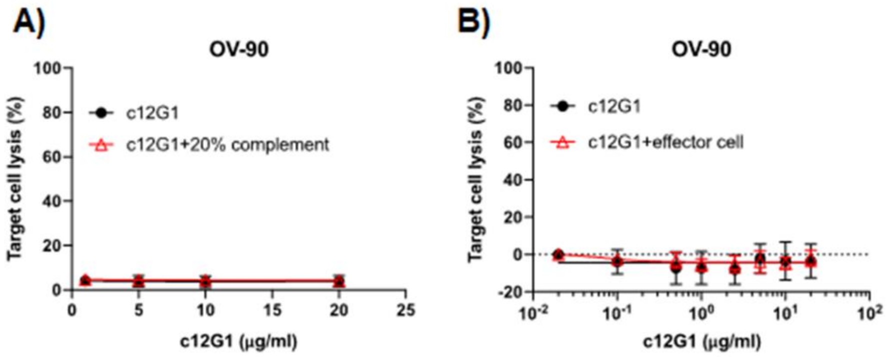

Figure S2. *In vitro* CDC and ADCC analyses of c12G1 antibody. (A) CDC activity was examined using OV-90 cells as indicated in the presence of absence of 20% human serum complement (v/v) for 6 h. (B) ADCC activity was examined using OV-90 cells in the presence of human PBMC (effector/target ratio = 30:1) with the indicated concentration of 12G1 antibody for 6 h. Then, cells were directly counted using Hoechst 33342 staining. The results represent the means ± SD of three independent experiments.

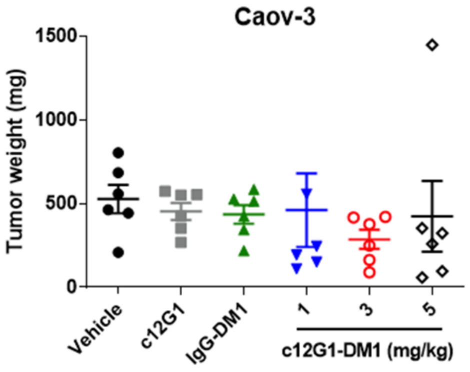

Figure S3. Evaluation of tumor volume. At day 41, tumor volume of vehicle, c12G1 antibody, IgG-DM1, or c12G1-DM1 administered mice implanted with Caov-3 cancer cells was calculated and individually plotted.

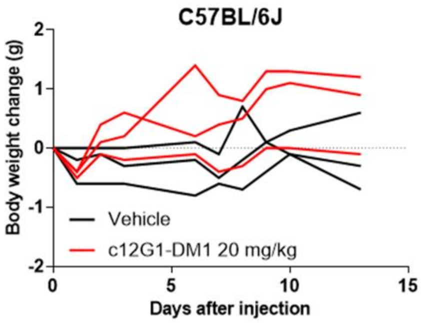

Figure S4. Single toxicity analysis of c12G1-DM1 in mice. The vehicle or c12G1-DM1 was intravenously administered to normal C57BL/6 mice (n=3). The change of body weight was monitored for 13 days.

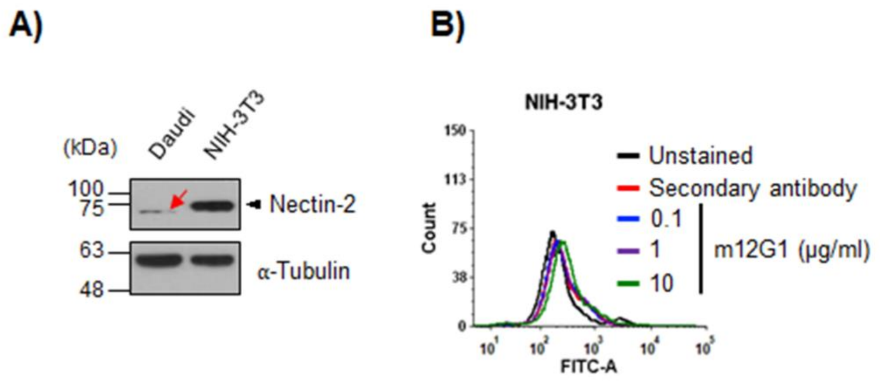

Figure S5. Species cross reactivity analysis of m12G1 antibody. (A) The expression of nectin-2 was examined in NIH-3T3 cell line, mouse embryonic fibroblast. Daudi was used as a nectin-2 negative cell line and tubulin was used as a loading control. The red arrow indicates a non-specific band. (B) The binding of m12G1 antibody, targeting human nectin-2, to NIH-3T3 cell expressing nectin-2 was examined in various concentration of m12G1 antibody by flow cytometry.

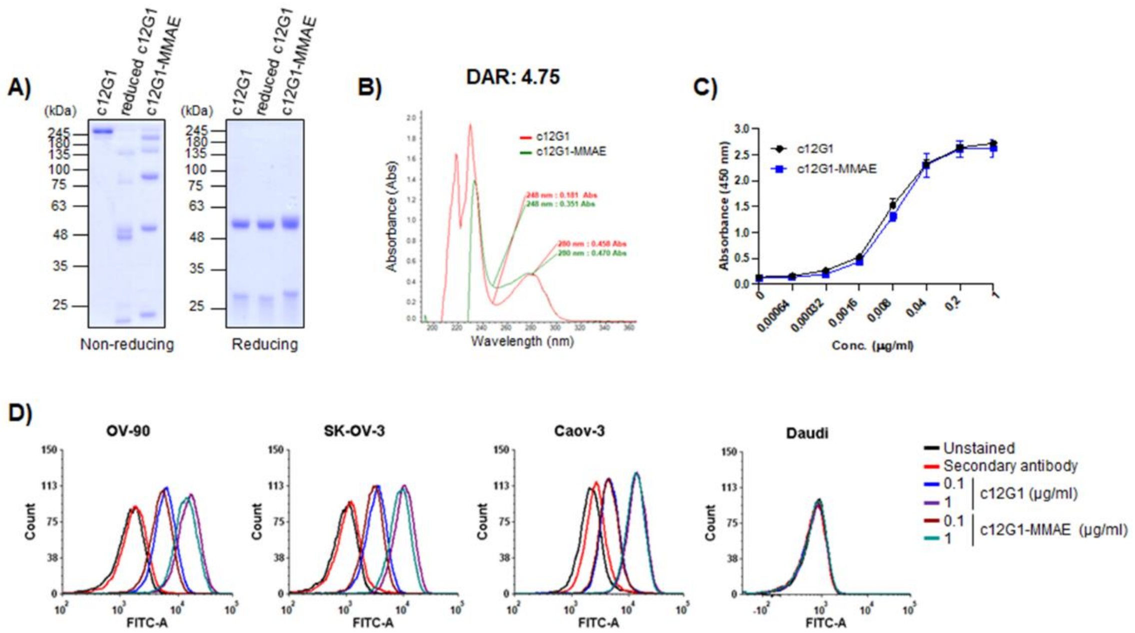

Figure S6. Characterization of c12G1-MMAE. (A) The naked intact c12G1 antibody, TCEP-reduced c12G1 antibody, and c12G1-MMAE were compared using non-reducing and reducing SDS-PAGE. (B) Optical absorbance at 248 nm of c12G1-MMAE was compared with that of naked c12G1 antibody. The calculated DAR was determined as 4.75. The binding affinity of naked c12G1 antibody and c12G1-DM1 to human nectin-2 protein was compared using ELISA (C) and flow cytometry (D). Daudi cells were used as a nectin-2 negative cell line. The nectin-2 binding affinities of naked c12G1 antibody and c12G1-MMAE were similar. All experiments were independently repeated at least three times.

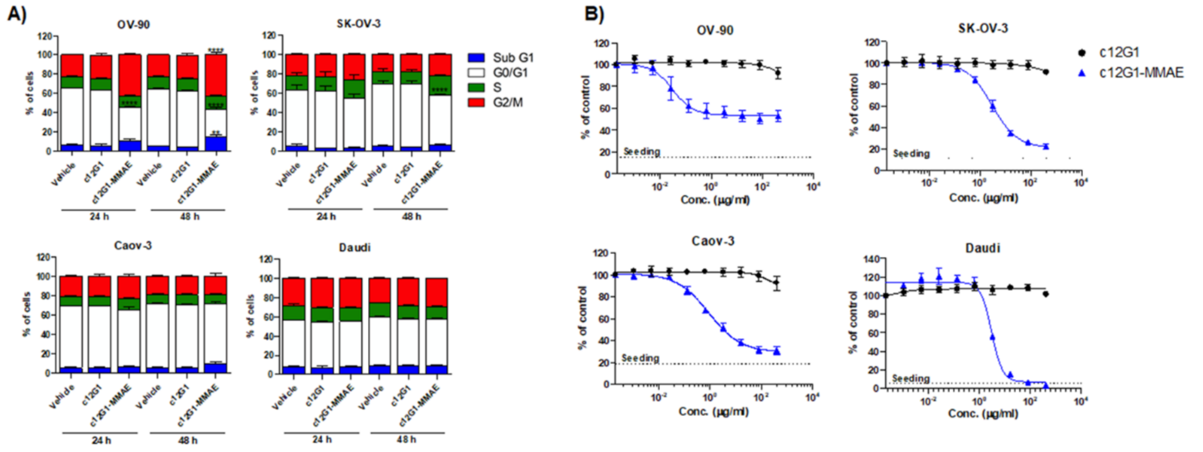

Figure S7. c12G1-MMAE exhibits antitumor activity. (A) Ovarian cancer cells were treated with vehicle, c12G1 antibody (1 μg/ml), or c12G1-MMAE (1 μg/ml) for 24 h and 48 h. Then, the cells were fixed and stained with propidium iodide followed by cell cycle analysis using a Celigo Imaging Cytometer (**, and **** vs. their respective corresponding vehicle and c12G1 antibody). c12G1-MMAE increased cell population of G2/M phase at 24 h followed by an increase of sub G1 population at 48 h. Daudi cells were used as nectin-2 negative cell line. The results represent the mean ± SD from at least three independent experiments. (B) Cells were treated with serially diluted concentration of c12G1 antibody or c12G1-MMAE for 3-4 days. Cells were stained with Hoechst 33342 (10 μM) at 37 °C for 30 min and quantitated using a Celigo Imaging Cytometer. The results represent the mean ± SD from at least three independent experiments. Dashed line indicates cell seeding number. ** $P < 0.01$ and **** $P < 0.0001$.
